# Student-to-Student: A Novel Approach to Community Outreach

**DOI:** 10.7759/cureus.64091

**Published:** 2024-07-08

**Authors:** Grant H McDaniel, Tricia Aho, Shirley Bodi, Carlos A C Baptista, Patrick W Frank

**Affiliations:** 1 Medical Education, University of Toledo College of Medicine and Life Sciences, Toledo, USA; 2 Family Medicine, University of Toledo College of Medicine and Life Sciences, Toledo, USA; 3 Neurobiology and Anatomy, Drexel University College of Medicine, Philadelphia, USA

**Keywords:** community outreach, innovation, leadership, medical education, mentorship

## Abstract

Community outreach is an established method for organizations to interact with the community. It is often done to help improve the community and its members by providing resources and educational opportunities. A growing crisis facing the United States of America is a worsening physician shortage, which will negatively impact many across the nation, especially vulnerable communities. The Student-to-Student organization offers a novel approach to community outreach by helping introduce and inspire high school students and young adults to pursue medicine. The organization is run by medical students and supervised by the College of Medicine faculty. It offers students from local high schools, community colleges, and undergraduate universities the opportunity to visit the medical college, where they can explore human anatomy and discuss the different facets of medicine and medical education with current medical students. This experience provides medical students with the ability to refine their public speaking abilities, gain leadership experience, improve their professional identity, and be involved in meaningful community outreach. These attributes also serve to enhance medical student residency applications at a time when uncertainties abound in the aftermath of Step One becoming pass/fail. The organization has run continuously since 1987 (except for one year during the COVID-19 pandemic). It has evolved over the years to become a high-performing organization that provides more than 80 tours yearly to thousands of students from the surrounding communities. This article aims to provide a detailed description of the history, organization, and impact of the Student-to-Student organization so that other medical students have a framework for implementing a similar program at their institution.

## Introduction and background

Local communities are the foundation on which societies are built. A community’s ability or lack thereof to produce goods, services (i.e., healthcare), culture, intellectuals, etc. can directly impact the community members’ well-being, surrounding communities, and society. That is why various organizations and individuals engage in some form of community outreach and investment. Community outreach has been described simply as “establish beneficial connections between people and organizations” [1]. Organizations ranging from wealthy individuals, professional sports teams, businesses, governments, and countless others donate billions of US dollars each year to smaller organizations worldwide. Funding from these organizations benefits smaller community-based endeavors, which consist of education institutes, religious organizations, local nonprofits, after-school athletic programs, and many more. The overall goal of community outreach is to provide resources and opportunities for individuals to improve themselves and ultimately their community.

Communities in the United States of America (USA) are currently facing a crisis that is only projected to get worse, a crisis resulting from a shortage of physicians. Organizations like the American Medical Association (AMA) and the United States government have funded and produced research along with legislative options to determine the cause, impact, and practical solutions to this worsening crisis [2,3]. By the year 2030, it is estimated that there will be a physician deficit of 139,160 [4]. This deficit was further evaluated by a 2017 report from the AMA that indicates there will be a physician deficit of 21,000-55,000 in primary care, 17,000-29,000 in surgical specialties, and 9,000-19,000 in medical specialties by 2033 [5]. The physician shortage is multifactorial owing in part to burnout, an aging workforce, a decreasing level of autonomy, and increased demands from complex electronic health records to name a few [6]. This lack of physicians already directly impacts the well-being of communities, and if unaddressed, will just be more devastating.

Becoming a physician is a long and complex process, which to someone with no touchpoint to medicine, could seem next to impossible. In summary, it involves multiple application processes, a myriad of examinations (i.e., entrance, university, and national boards), hundreds of thousands of dollars, greater than a decade of your life, and a commitment to lifelong learning [5]. Having a family member who is a physician or in health care is extremely helpful. However, the latest statistics from AAMC estimate that 12.4% of medical doctorate students are first-generation college graduates [7]. There is a growing realization of the need for diversity in medical school classes, which will enable future physicians to relate to and care for the communities they serve. The goal is to recruit classes made up of students from all different races, ethnicity, gender identity, socioeconomic status, geographic background, and students with disabilities [8]. Providing community outreach to introduce high school students and young adults to human anatomy, medical education, and the career of medicine, has the potential to inspire them to pursue medicine. This outreach can be especially beneficial for those from diverse backgrounds or those with no touchpoint with medicine. While a community outreach endeavor like this will not solve the physician shortage crisis, it can be one of many useful tools employed to combat this worsening healthcare and community crisis.

Student-to-Student overview

An open-access website search of all medical schools accredited by the Liaison Committee of Medical Education (LCME) and the American Association of Colleges of Osteopathic Medicine (AACOM) was performed [9,10]. The LCME and AACOM have 168 and 37 medical colleges, respectively. Each website was searched for the terms “anatomy lab tour” and “student-led anatomy lab tour.” This search yielded 15 schools, in addition to the University of Toledo College of Medicine and Life Sciences, that offer tours of the anatomy lab (tours for recruitment during medical school application and/or alumni events were excluded). Further investigation revealed that of the 16 programs, only 5 provide medical student-led tours and only one offered hands-on tours of the anatomy lab and human cadavers on a regular basis, free of charge. This indicates that the University of Toledo College of Medicine and Life Sciences Student-to-Student (S2S) program provides a unique opportunity for student tour guides and student participants alike.

Student-to-Student is a medical student-run, faculty-supervised organization that provides medical students with the ability to volunteer, teach, lead, and offer mentorship all while providing meaningful community outreach. The organization facilitates interactive tours of the anatomy lab and the on-campus human anatomy plastination museum to local high schools, community colleges, undergraduate students, emergency medical technician programs, and many more. The following sections will explain in detail the inception and history of the program, the organization, and the impact the organization has had. The aim is to provide a model for other medical schools wishing to implement this novel program.

History

Student-to-Student was started in 1986 by medical students with the idea of improving public health and providing high school and college students with the ability to learn about medicine and medical school. The founding medical students drafted a letter that they sent to local schools advertising the organization and what services they offered. Originally, they would offer discussions about the physiology and pathology of the respiratory, cardiovascular, nervous, renal, and hepatic systems. Students with permission from the school would bring certain cadaveric specimens to teach anatomy and demonstrate pathology, for example, bringing an emphysema lung and a healthy lung to demonstrate the effects of smoking. Then, medical student Shirley Bodi joined the organization in 1988 and currently serves as one of the faculty advisors. She helped develop a public health initiative entitled “going to the doctor”; this was tailored to kindergarteners and first-grade students to teach them why they go to the doctor and what happens at the doctor’s office by explaining the various tools used during the physical exam. During Dr. Bodi’s tenure, she reached out to a new anatomy faculty member, Dr. Carlos Baptista, and forged a partnership that is still active today.

In 1987, Dr. Baptista became involved in the plastination of cadaveric specimens for Student-to-Student, which facilitated a cleaner and more efficient mechanism for teaching anatomy. He subsequently became the faculty advisor for the organization. In the early 90s, the organization was so popular with schools in the community and placed increasing demands on the volunteer students that the College of Medicine had to step in. The college decided that tours would now primarily take place on campus in the anatomy lab during the medical students’ lunch hour. Medical students continued providing tours with more of a focus on human anatomy and occasionally visiting schools to give presentations. Dr. Baptista also started to hold educational sessions on the plastination process for medical students and tour groups. With the large amount of plastination work taking place, the Liberato Dido & Peter Goldblatt Interactive Museum of Anatomy and Pathology was created. The museum consists of both normal and pathological plastination of human anatomy. Dr. Baptista retired in 2019, leaving the faculty leadership of the organization to Dr. Patrick Frank. Dr. Frank continued to work with Dr. Baptista to curate anatomical specimens for the lab and helped lead the organization through the COVID-19 pandemic and its aftermath. Dr. Frank also helped modernize the organization by overseeing the development of a website for tour registration. Additionally, he spent years designing, advocating, and overseeing an elective course for students who participated in the Student-to-Student organization approved by the College of Medicine. Drs. Bodi and Baptista still serve as faculty advisors to the organization today.

Organization 

The modern-day Student-to-Student organization consists of volunteer first- through fourth-year medical students. Second-year medical students have the opportunity to serve in leadership roles on the executive board for one year as either the President, Vice President of Operations, Vice President of Public Relations, Secretary, or Lab Coordinator. In addition to giving tours, the executive board oversees caring for the donor body, performing dissections, public relations, training new tour guides, and managing the logistical demands of the organization. All actions of Student-to-Student are coordinated through the President and Faculty advisors. For medical students to provide tours, they are required to observe one tour and then give a tour proctored by one of the executive board members.

Tours are offered once per day during the medical students' lunch hour, tours are scheduled via utcoms2s.org. Utcoms2s.org is a website that was designed, developed, and maintained by John Ziebro who at the time was a medical student member of Student-to-Student. On the front end, the website allowed the organization to have a centralized location for visiting schools to learn about the opportunities offered and the requirements for visiting, and it provided a more efficient and organized manner of scheduling. On the back end, the website allowed for the recording of organization metrics that provided a quantitative method for measuring impact. This website is also utilized by the tour guides to sign up for tours. When organizations sign up, they are automatically sent a visitor contract and lab etiquette document; a signed copy is required from each visitor on the day of their tour. The tours themselves are limited to a maximum of 40 visitors at a time, with one tour guide per 20 students. Any tour with over 20 students is split into two groups to allow one tour guide to start in the Plastination Museum and another tour guide to start in the anatomy lab. Approximately 30 minutes into the tour, the groups switch locations. Additionally, a third tour guide is utilized if available and the tour group is split into three groups. Because tours are offered to multiple educational levels, and at various times of the school year, tour guides tailor the depth of information they provide to the level of the students participating in the tour. The content of tours may also be adjusted according to the interest of the visiting students (e.g., tailoring the tour to a paramedic class vs to a high school anatomy class). Tour guides, if not asked specific questions, will talk with visitors about topics like how to get into medical school, what the medical school experience is like, and different areas and specialties of medicine. There have been several students able to offer a unique perspective on the Student-to-Student organization because they had received tours prior to attending medical school and then became tour guides themselves. The Student-to-Student organization has been impactful for community outreach by providing a means for medical students to teach and potentially inspiring a substantial number of students interested in medicine from a diverse geographical catchment.

## Review

**Figure 1:**
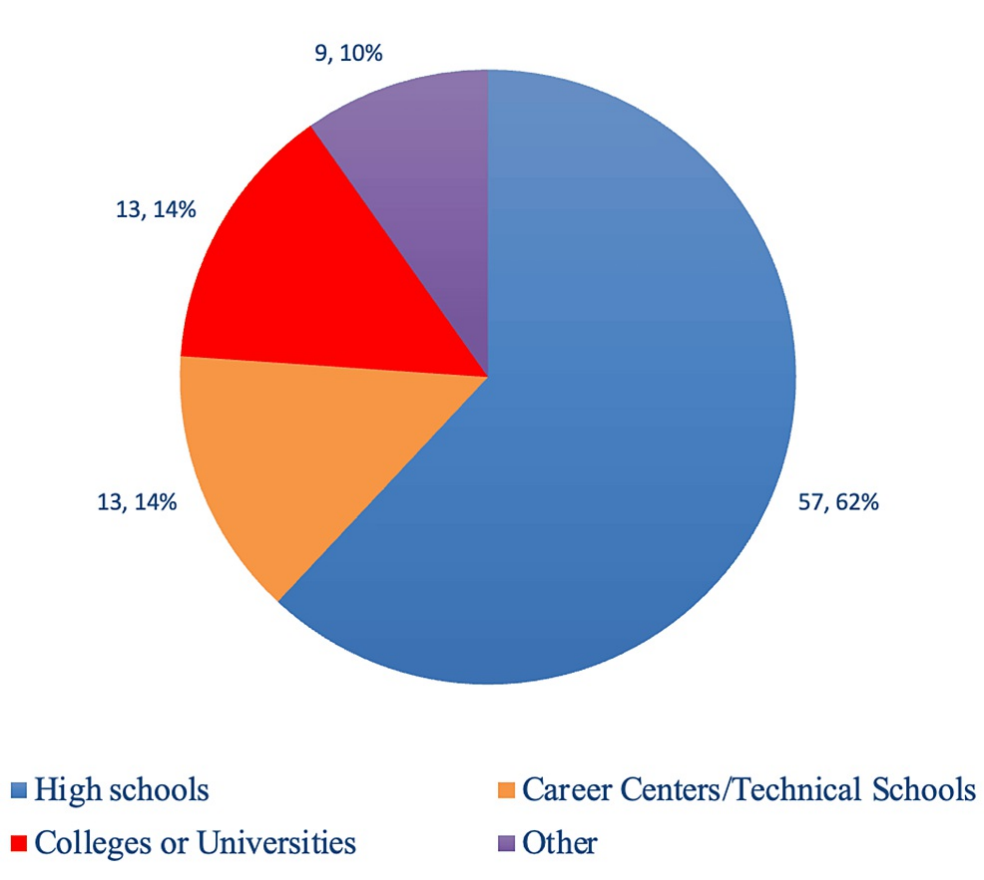
Breakdown of visiting program types

**Figure 2:**
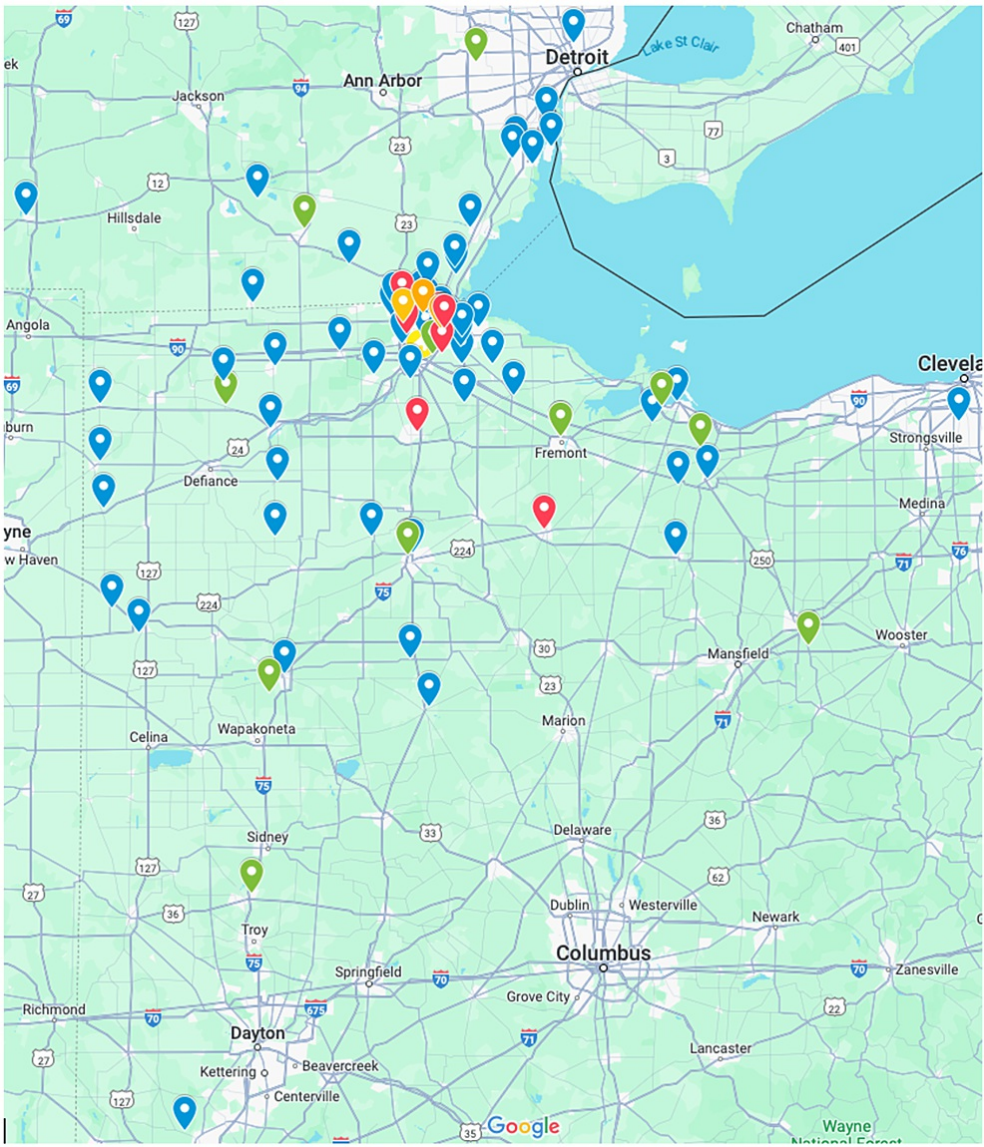
Geographic catchment of visiting programs (generated via Google Maps) Blue marker: high school; Green marker: career centers or technical school; Red marker: college or university; Yellow marker: University of Toledo College of Medicine and Life Sciences

There are currently 88 organizations that are registered on the website to sign up for tours. Of these 88 organizations, 57 are high schools, 13 are career centers/technical schools, 9 are colleges or universities, and 9 are various other organizations (Figures 1, 2.). Many of these visiting organizations come from Northwest Ohio and Southeast Michigan. However, there is a wide-reaching geographic catchment area with organizations from more than 10 countries. The farthest north and south that organizations have traveled for tours are approximately 71 and 180 miles, respectively (Figure 2). Since recordkeeping started with the implementation of the website, Student-to-Student has given 262 tours to 6,042 students (Table 1).

**Table 1:** Number of tours given per year

Academic Year	Number of Tours	Number of Students
2020-2021	26	810
2021-2022	101	2,302
2022 -2023	91	1,987
2023-2024 (As of 2/17/2024)	44	943

Student-to-Student has had a significant impact both for medical students, faculty, and the community. Since its inception, Student-to-Student has had hundreds of medical students involved, with each year having 30-40 active tour guides. As stated above these tour guides range from first to fourth-year medical students. This provides a unique opportunity for the more senior medical students to teach their junior counterparts (a continuing theme throughout residency and when they are attending). Initially, the senior students share their knowledge of anatomy and how to be an engaging teacher/public speaker. This provides the junior students with the foundation to provide impactful tours to visitors. For those medical students who become more involved, the Executive Board seniors have the opportunity to teach their juniors how to lead, adapt, and communicate in a fast-moving organization. Additionally, they teach how to interface professionally with other educators and educational institutions. Outside of organizational roles and responsibilities, senior medical students are able to serve as both a resource and a mentor for junior medical students, providing them with an informal avenue for addressing educational concerns, advising on rotations, and even answering residency application questions. The recent decision to make Step One the first of three tests required by the United States Medical License Examination pass-fail has left medical students, schools, and residents wondering what will take its place. Speculations abound about what will be substituted for Step One, but the data are not yet available to determine exactly what that will be. Some speculate that there will be more emphasis placed on extracurricular and leadership experience. Student-to-Student offers a unique and efficient way to bolster a medical student's residency application in multiple ways.

Faculty oversight is essential to ensure all the above occurs and that there is continuity of standards from year to year. Additionally, faculty closely mentor and advise the executive board students, helping them navigate and problem-solve organizational issues that arise. The faculty advisor, along with the President, oversees an elective program that does show up on the official transcript of those tour guides who give more than eight tours per year. The whole organization provides a positive feedback loop for mentorship along with personal and professional growth for all parties involved. Equally, if not more, impactful than the experience of the students and faculty is the effect on the community.

## Conclusions

Community outreach is vitally important to strengthening communities and their members. The United States of America is facing a growing crisis because of a worsening physician shortage, which will negatively impact many communities across the country. This is a multifaceted problem that will require not just one but many solutions. Student-to-Student is a novel medical student-run, faculty-supervised organization that provides dozens of tours to hundreds if not thousands of students from diverse backgrounds per year. These students are offered the unparalleled opportunity to study human anatomy and gain exposure to medicine with the hopes of inspiring them to pursue medicine as a career. Additionally, it provides medical students with the ability to refine their public speaking abilities, gain leadership experience, improve their professional identity, and be involved in meaningful community outreach. All of these factors also help strengthen medical students’ residency applications at a time when many are wondering what will take the place of Step One. Student-to-Student can serve as a model for other medical schools to follow to help improve and strengthen their relationship with their local community, with the added goal of being a useful tool to help mitigate the growing physician shortage by inspiring the next generation of physicians.
